# Magnetic Resonance Imaging Systems Equipment Hardware Today and Tomorrow

**Published:** 1988-05

**Authors:** Derek C. Beatty

**Affiliations:** Marketing Manager, MRI, Picker International Ltd


### Magnetic Resonance Imaging Systems Equipment Hardware Today and Tomorrow

**Derek C. Beatty BSC Dip M,** Marketing Manager, MRI, Picker International Ltd

MR is undoubtedly the most revolutionary imaging technology of the century. The clinical usefulness of MRI has been widely accepted in the CNS, spine and certain body areas and the future vision of MR includes wide-ranging morphological and functional applications in cardiology, oncology, orthopaedics, obstetrics and sports medicine. In order to accommodate this wide range of clinical applications, Picker has developed the Vista MR2055 HP system which delivers maximum clinical imaging flexibility at field strengths from 0.5 Tesla to 1.5 Tesla and spectroscopic capability at 1.5 or 2.0 Tesla. Five primary subsystems are integrated to produce an MR imager capable of high resolution proton images in axial, coronal, sagittal or oblique planes. These primary subsystems include the magnet, gradient, radiofrequency and control subsystems which in turn are driven by a powerful, multi-tasking computer.

*Magnet*: Superconductive magnet options include field strength variability between 0.5T and 2.0 Tesla. The magnet design provides a high degree of uniformity during imaging over the imaging volume. Superconducting niobium titanium windings are immersed in liquid helium and thermally insulated by liquid nitrogen to maintain a stable operating temperature of 4.2° Kelvin. Electronically adjusted cryogenic and resistive shim coils maintain field uniformity during imaging sequences. When the magnet is at field liquid nitrogen boils off at 1.0 litre/hour and liquid helium at 0.5 litre/hour. The wide bore of today's magnets, 60 cm in the centre of the magnet, minimises claustrophobic effects. New active shield magnets reduce the fringe magnetic field by up to 95% and enable MR systems to be sited in locations previously thought impossible.

*Patient Couch*: The patient couch is capable of both horizontal and vertical movement to facilitate easy patient loading and unloading. Patient positioning is by laser index and a single operator can easily manage patients of any size in or out of the static field. A decorative facade surrounds the magnet to make the environment pleasing to the patient. Safety features for the patient include a hand activated patient alarm, two way voice communication between the patient and the operator at the console, magnet bore illumination, ventilation and visual indications for cardiac and respiratory monitoring.

*Gradient Subsystem*: The X, Y and Z multicore gradient coils are located between the shim and patient tube and a special mounting minimises acoustic noise. Gradient coils are designed to allow the best combination of linearity, switching rate and gradient strength to provide a wide range of technique flexibility. Typical gradient field strength is 6.5 mT/m going up to 11 mT/m with a rise time of less than 1 m sec. This power enables slices as thin as 2 mm to be taken in transverse, sagittal, coronal and oblique planes utilizing direct excitation. Imager tuning is performed automatically, prior to the study from the patient console and can be to standard or specific protocols.

*Low Level R.F. System*: This consists of a synthesizer, transmitter and receiver. The transmitter section generates resonant frequency with preset phase, amplitude, shape and duration at a lower power level. The real and imaginary quadrature receiver section of the system transforms the MR signal into a digital representation. The Low Level RF System is computer controlled.

*Radio Frequency Subsystem*: The radio frequency coil set acts as the transmitter of RF energy and receiver of MR signals. Coils are automatically tuned by the system to ensure optimum signal reception for each patient. R.F. deposition is continuously monitored to ensure patient safety. Head and body coils are the main RF coils used and are designed to give good homogeneity over a wide field using minimum power. Speciality surface coils have been designed to image the cervical spine, lumbar spine, joints, orbits and ears and provide image quality improvement for small volume, specific area MR imaging. Speciality coils act as receivers which maximize signal reception by closely conforming to the specific body part being imaged.

*Computer System*: True multi-tasking system functions are controlled by the 32 bit computer. Advanced system software provides high speed capability to perform scanning, image display and filming from local or remote systems simultaneously. The computer includes a random access memory, floating pont array processor, disk drive, a magnetic tape system for long-term data storage and a hardcopy recorder. Options to the computer system include a line printer, optical disk archival system and data communications link to a stand alone viewing system which in turn can be linked by its own data communications link to another MR system or a CT system. The computer system also provides for preset sequences and easy programming for new sequences.

*Diagnostic Console*: The operator/viewer console's unique design affords operator ease of use and complete control of all procedural and imaging variables. The software design easily adapts to clinical and research applications using MR. The main console unit consists of a standardised keyboard, intercom system, image monitor and text monitor. The independent station enables simultaneous scan and display functions for one or two operators and multiple scans can be run without further operator interaction. The standardised keyboard is equipped with special function keys to provide initiation of routine proctocols and also a stack capability which can provide a fast review of up to 64 image studies. A two way intercom allows operator/patient communication throughout the examination.

**Conclusion**

The MR systems developed by Picker International provide the MR technology requirement for MR clinical and research applications not only to meet today's imaging needs but to provide a practical long term choice. These systems serve the widest range of requirements when consideration is given to total performance, clinical utility, economics, technical standards and environmental considerations.

**The Future**

There are over 1,200 MRI systems installed worldwide compared to over 7,000 CT scanners. MRI has the potential to replace up to 60% of the workload of CT due to the clinical benefits which it can provide over CT. New applications in cardiology, sports medicine and other functional areas could lead to the development of MRI market growth into specific market segments. Capital funding of equipment in health services worldwide and private insurance reimbursement rates are the main constraints to the growth of the MRI Market in USA, Europe and Japan. If MRI is to be purchased for extensive use as an imaging modality in district general and private hospitals, it has to be shown as cost effective compared to alternative procedures. This is not straightforward since savings in healthcare costs, for example due to early diagnosis or outpatient examinations, are not generally identified and set against the higher capital costs of advanced techniques such as CT and MRI. It may be possible with new technology to develop systems of a lower cost and performance which are adequate for specific clinical applications. Another way to reduce cost may be in specific innovations, such as high temperature superconductors in magnet construction. The use of new high temperature superconductors in magnet construction could lead to a slight reduction in magnet costs but by switching from liquid helium to liquid nitrogen the cost of running an MRI scanner could be reduced significantly. The medical application of magnets for MR scanners is only one application of the use of superconductors and it could be several years before new developments in superconductivity emerge as being beneficial to MRI scanners.

In view of the timescale and uncertainty of such innovations, it is likely that for the foreseeable future users will require all the facilities of current high performance systems, to handle the varied case load of a general hospital.

